# Monitoring a Mystery: The Unknown Right Ventricle during Left Ventricular Unloading with Impella in Patients with Cardiogenic Shock

**DOI:** 10.3390/jcm13051265

**Published:** 2024-02-23

**Authors:** Birgit Markus, Julian Kreutz, Giorgios Chatzis, Styliani Syntila, Maryana Choukeir, Bernhard Schieffer, Nikolaos Patsalis

**Affiliations:** Department of Cardiology, Angiology, and Intensive Care Medicine, University Hospital, Philipps University of Marburg, 35037 Marburg, Germany; kreutzj@staff.uni-marburg.de (J.K.); chatzis@staff.uni-marburg.de (G.C.); syntila@med.uni-marburg.de (S.S.); choukeir@staff.uni-marburg.de (M.C.); bernhard.schieffer@staff.uni-marburg.de (B.S.); patsalis@med.uni-marburg.de (N.P.)

**Keywords:** cardiogenic shock, left ventricular Impella, right ventricular function, predictors of RV function

## Abstract

**Background**: Right ventricular (RV) dysfunction or failure occurs in more than 30% of patients in cardiogenic shock (CS). However, the importance of timely diagnosis of prognostically relevant impairment of RV function is often underestimated. Moreover, data regarding the impact of mechanical circulatory support like the Impella on RV function are rare. Here, we investigated the effects of the left ventricular (LV) Impella on RV function. Moreover, we aimed to identify the most optimal and the earliest applicable parameter for bedside monitoring of RV function by comparing the predictive abilities of three common RV function parameters: the pulmonary artery pulsatility index (PAPi), the ratio of right atrial pressure to pulmonary capillary wedge pressure (RA/PCWP), and the right ventricular stroke work index (RVSWI). **Methods**: The data of 50 patients with CS complicating myocardial infarction, supported with different flow levels of LV Impella, were retrospectively analyzed. **Results:** Enhancing Impella flow (1.5 to 2.5 L/min ± 0.4 L/min) did not lead to a significant variation in PAPi (*p* = 0.717), RA/PCWP (*p* = 0.601), or RVSWI (*p* = 0.608), indicating no additional burden for the RV. PAPi revealed the best ability to connect RV function with global hemodynamic parameters, i.e., cardiac index (CI; *p* < 0.001, 95% CI: 0.181–0.663), pulmonary capillary wedge pressure (PCWP; *p* = 0.005, 95% CI: −6.721–−1.26), central venous pressure (CVP; *p* < 0.001, 95% CI: −7.89–5.575), and indicators of tissue perfusion (central venous oxygen saturation (SvO_2_); *p* = 0.008, 95% CI: 1.096–7.196). **Conclusions:** LV Impella does not impair RV function. Moreover, PAPi seems to be to the most effective and valid predictor for early bedside monitoring of RV function.

## 1. Introduction

Right ventricular (RV) dysfunction or failure is an often unrecognized and underestimated problem in cardiogenic shock (CS) complicating acute myocardial infarction. According to the current literature, more than 30% of the patients suffering from CS are affected [1,2]. It occurs regardless of the culprit lesion, depends on various pathologies, and is associated with increased mortality. Thus, early detection and treatment of RV failure is crucial for patient management and outcome.

Among others, RV dysfunction is a feared condition during therapy with long-term LV assist devices (LVAD) [3,4,5]. The pathophysiology of right ventricular failure during LVAD therapy remains incompletely characterized and seems to be multifactorial. Post-surgical changes of myocardial geometrics, resulting in alterations of ventricular interdependence and thus hemodynamics, have been considered to play a significant role in right ventricular dysfunction or failure following LVAD implantation [6,7].

Basically, LVAD generates a continuous laminar blood flow between the LV and the aorta ascendens, thus unloading the LV. During acute left ventricular unloading with LVAD, the right ventricular preload increases due to increased cardiac output. In this situation, right ventricular contractility and function depend on the functional myocardial reserve of the RV. If right ventricular dysfunction or failure occurs during LVAD therapy, RV volume overload leads to a leftward septal shift, resulting in left ventricular compression. If the left ventricular geometry changes this way, left ventricular ejection through the native aortic valve becomes more difficult, which in turn causes an increase in RV afterload. In the case of pre-existing aortic valve insufficiency, the regurgitation volume may lead to an additional increase in RV afterload, further deteriorating RV function [8]. Moreover, in the event of pump suction due to actual or relative hypovolemia, a septal shift to the left side occurs. The resulting variation of the RV morphology causes an impairment in RV contractility, ultimately leading to RV dysfunction and failure. This may occur, among others, due to increased RV afterload. In addition, it has to be mentioned that due to the existing ventricular interdependence, the quality of the RV function fundamentally depends on the LV function and vice versa [9,10].

Nowadays, percutaneously implanted temporary MCS like the Impella are increasingly used for hemodynamic support and ventricular unloading in CS [11,12]. However, data on the impact of LV mechanical assist devices like the Impella on RV function are rare [13,14]. To ensure the most optimal performance of the LV Impella, avoiding suction or hemolysis, the RV function and the volume status of the patient are of great interest.

Parameters for bedside monitoring and evaluation of the RV function during LV Impella support are differentially discussed in the current literature [15,16]. Commonly used parameters for monitoring the RV function are the right ventricular stroke work index (RVSWI) and the ratio of right atrial pressure to pulmonary capillary wedge pressure (RA/PCWP). Another new and easy-to-monitor hemodynamic predictor of RV function is the so-called pulmonary artery pulsatility index (PAPi). PAPi is calculated as the ratio of the pulmonary artery systolic pressure minus the pulmonary artery diastolic pressure divided by the right atrial pressure. As described in the literature, lower PAPi values (<2) indicate a compromised RV function and are associated with greater mortality, major adverse cardiac events (MACE), and hospitalizations due to heart failure [17,18]. Recently, PAPi was confirmed as a predictor for RV failure in patients with ongoing long-term LVAD therapy [19,20].

We here evaluated the effect of LV support and unloading with the Impella on the RV function by demonstrating the consequences of biventricular interference and comparing the aforementioned RV performance indices. The aim was to define the most optimal predictor of RV function in patients with ongoing Impella support in CS during daily clinical routine.

## 2. Materials and Methods

### 2.1. Study Design

This is a retrospective analysis of additional collected data from a clinical single-center trial performed from January 2020 to February 2022 at the University Hospital of Marburg Germany. The study population consisted of 50 patients with CS complicating myocardial infarction treated with a left ventricular Impella (CP). The primary focus of the initial trial (2020–2022) was to evaluate the impact of Impella support on renal organ perfusion. These data have already been published [21]. In this retrospective sub-analysis of further documented data, the impact of LV unloading with the Impella on the RV function was investigated.

Inclusion criteria of the initial trial consisted of age above 18 years, signed informed consent, and CS complicating myocardial infarction. Patients with unstable hemodynamics were excluded, and according to the initial focus of the trial, those with only one kidney, underlying autoimmune issues, or polycystic kidney disease were excluded.

The definition of CS was (a) compromised systolic blood pressure of less than 90 mmHg for more than 30 min or the need for vasopressor/inotropic therapy to maintain systolic blood pressure >90 mmHg, (b) the presence of pulmonary congestion, and (c) clinical signs of insufficient end-organ perfusion (at least one of the following: pathological mental status, cold and wet skin, urine production <30 mL/hour, serum lactate >2.0 mmol/L).

Impella implantation was performed within 120 min following myocardial infarction and admission to the hospital. The implantation of the Impella as well as of the pulmonary artery catheter (PAC) for invasive hemodynamic measurements was conducted according to established standard operating procedures in the catheterization laboratory before starting the PCI procedure.

Twenty-four hours after admitting the patients to the intensive care unit (ICU) and before the study-associated measurements started, the correct position of the Impella pump and a sufficient ventricular volume load were verified by echocardiography. Furthermore, the stability of hemodynamic parameters had to be documented for at least 6 h, since catecholamine dosages and fluid substitution needed to remain without alterations to avoid pathophysiological influences on the measurements.

The first measurement of hemodynamic parameters during Impella support depicted baseline values indicating a relatively low Impella performance level. In order to confirm hemodynamic stability, a second measurement followed 60 min later. Thereafter, while the Impella output was changed, further treatment-related parameters, like blood pressure, catecholamine dosages, and fluid substitution, remained without any alterations (Table 1 and Table 2). Afterward, the Impella support was enhanced by approximately 0.5 L/min. After the hemodynamic changes had presumably returned to a stable state following the change in the Impella flow rate, the third measurement during the increased Impella flow was carried out 30 min later. Next, the Impella output was once again elevated by 0.5 L/min (1.0 L/min above baseline). Another 30 min later, hemodynamic parameters were measured for the fourth time.

### 2.2. Invasive Hemodynamic Measurement (Pulmonary Artery Catheterization)

Hemodynamic parameters like the cardiac index (CI), pulmonary capillary wedge pressure (PCWP), central venous oxygen saturation (SvO_2_), systemic vascular resistance (SVR), right ventricular stroke work index (RVSWI), central venous pressure (CVP), left ventricular stoke work index (LVSWI), systolic arterial pulmonary pressure (SPAP), diastolic arterial pulmonary pressure (DPAP), and mean arterial pulmonary pressure (PAM) were invasively measured via PAC.

### 2.3. Statistical Analysis

The data are displayed as absolute variables and percentages (%) for categorical variables and according to the distribution of variables either as median with interquartile range (IQR: 25th–75th percentile) or mean with standard deviation. The Shapiro–Wilk and Pearson tests were implemented to assess normality. Due to skewed distribution, a natural _log_-transformation of PAPi, RVSWI, and RA/PCWP was conducted in order to obtain a normal distribution. Univariate ANOVA was conducted to evaluate the differences among the various conditions. A simple linear regression analysis was performed to investigate associations of _log_PAPi, _log_RVSWI, and _log_PCWP with hemodynamic and tissue perfusion parameters. Correlations between RV and left ventricular function indices were obtained by Spearman correlation.

The analyses were performed using SPSS 24 (IBM, New York, NY, USA) and GraphPad Prism 6.0 (GraphPad Software, San Diego, CA, USA). A two-sided *p*-value below 0.05 was considered statistically significant.

## 3. Results

### 3.1. Patient Cohort

In a total of 50 patients, hemodynamic measurements were performed on three different Impella levels, resulting in a total of 150 measurements. The demographics, baseline characteristics, and relevant comorbidities of the patients are shown in Table 1. All patients received coronary intervention and implantation of Impella CP via the femoral artery catheterization laboratory. When starting the measurements, the mean systolic ejection fraction was 37% ± 18. During the measurements, mean catecholamine dosages were 0.12 ± 0.19 µg/kg/min norepinephrine and 3.1 ± 2.67 µg/kg/min dobutamine, depicting the variance in the severity of cardiogenic shock of the study population. Nine patients had a history of chronic obstructive pulmonary disease (COPD) but without any relevant pulmonary arterial hypertension. During 30-day follow-up, 9/50 patients (18%) died. In all patients, the Impella could be routinely weaned and explanted. No patient received Impella therapy as a bridging strategy to LVAD or transplantation.

### 3.2. Associations between _log_PAPi, Hemodynamics, and Tissue Perfusion

**Figure 1:**
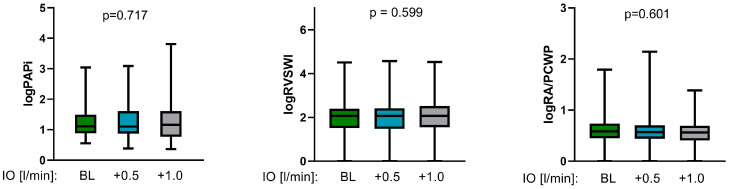
Impact of increasing Impella output (IO) on RV function indices (PAPi, RVSWI, RA/PCWP) (PAPi: Pulmonary Artery Pulsatility index, RVSWI: right ventricular stroke work index, RA/PCWP: right atrial pressure/pulmonary capillary wedge pressure).

**Figure 2:**
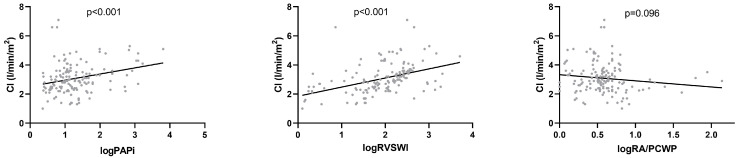
Linear regression analysis of CI with _log_PAPi, _log_RVSWI, and _log_RA/PCWP. CI: cardiac index, PAPi: Pulmonary Artery Pulsatility index, RVSWI: right ventricular stroke work index, RA/PCWP: right atrial pressure/pulmonary capillary wedge pressure.

**Figure 3:**
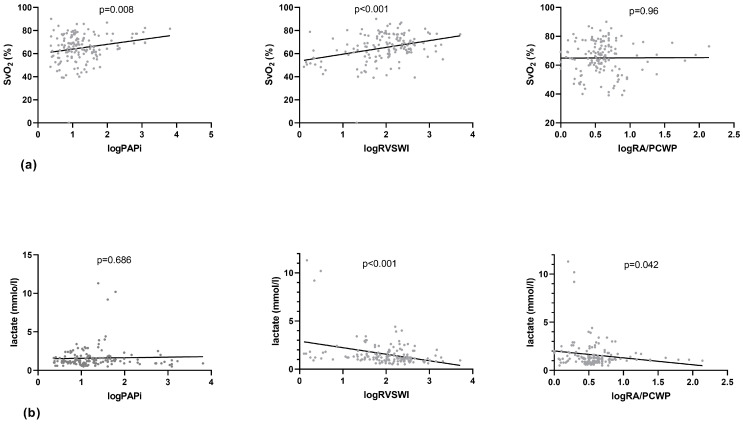
Linear regression analysis of perfusion markers with _log_PAPi, _log_RVSWI, and _log_RA/PCWP. (**a**) SvO_2_ and (**b**) lactate (PAPi: Pulmonary Artery Pulsatility index, RVSWI: right ventricular stroke work index, RA/PCWP: right atrial pressure/pulmonary capillary wedge pressure, SvO_2_: central venous oxygen saturation).

**Figure 4:**
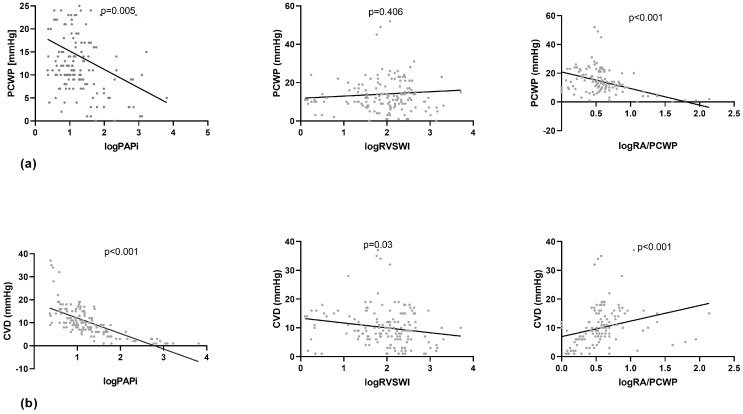
Linear regression analysis of congestion markers with logPAPi, logRVSWI, and logRA/PCWP. (**a**) PCWP and (**b**) CVD (PCWP: pulmonary capillary wedge pressure, CVD: central venous pressure, PAPi: Pulmonary Artery Pulsatility index, RVSWI: right ventricular stroke work index, RA/PCWP: right atrial pressure/pulmonary capillary wedge pressure).

Enhancing the Impella output by a mean of 0.5 L/min and 1.0 L/min did not lead to a significant alteration of _log_PAPi (Table 3, Figure 1), indicating no additional RV burden due to increased Impella output. However, the linear regression analysis presented a significant positive linear relationship of _log_PAPi with CI (*p* < 0.001, 95% CI: 0.181–0.663) and SvO_2_ (*p* = 0.008, 95% CI: 1.096–7.196) and a significant negative linear association with PCWP (*p* = 0.005, 95% CI: −6.721–−1.26) and CVP (*p* < 0.001, 95% CI: −7.89–5.575) (Table 4, Figure 2, Figure 3 and Figure 4). No significant correlation between _log_PAPi and lactate (*p* = 0.686, 95% CI: −0.276–0.419) was observed. These data indicate the ability of PAPi to reflect the influence of RV function on global hemodynamics and on parameters of tissue perfusion.

### 3.3. Associations between _log_RVSWI, Hemodynamics, and Tissue Perfusion

After augmenting the Impella output by a mean of 0.5 L/min and 1.0 L/min, _log_RVSWI showed no significant variations (Table 3, Figure 1). The linear regression analysis revealed a significant positive relationship of _log_RVSWI with CI (*p* = <0.001, 95% CI: 0.409–0.841) and SvO_2_ (*p* < 0.001, 95% CI: 3.21–8.55) and a significant negative relationship with CVD (*p* = 0.03, 95% CI: −3.19–−0.201) and lactate (*p* < 0.001, 95% CI: −0.992–−0.366) (Table 4, Figure 2, Figure 3 and Figure 4), whereas no significant association with PCWP (*p* = 0.406, 95% CI: −1.54–3.8) could be detected. These findings demonstrate the significant predictive ability of RVSWI regarding hemodynamics (CI, CVD) and tissue perfusion (SvO_2_, lactate). An increasing RVSWI, indicating improved RV function, is related to an enhanced CI and optimized tissue perfusion (SvO_2,_ lactate). However, in contrast to PAPi, RVSWI lacks the ability to reflect the interaction of RV function with post-capillary hemodynamic alterations and the affiliated LV congestion (PCWP).

### 3.4. Associations between _log_RA/PCWP, Hemodynamics, and Tissue Perfusion

Augmenting Impella output did not lead to significant variation of _log_RA/PCWP (Table 3, Figure 1). The linear regression analysis displayed a significant negative association of _log_RA/PCWP with PCWP (*p* = 0.096, 95% CI: −0.234–−0.104) and CVD (*p* < 0.001, 95% CI: 1.39–7.337) (Table 4, Figure 2, Figure 3 and Figure 4), whereas no significant associations with CI (*p* = 0.096, 95% CI: −0.234–−0.104) and SvO_2_ (*p* = 0.96, 95% CI: −4.317–7.551) could be demonstrated. Regarding lactate, a marginally significant negative association could be seen (*p* = 0.042, 95% CI: −1.618–−0.293). Although these data demonstrate the significant predictive ability of _log_RA/PCWP in CVD as a venous congestion parameter, the absence of a significant association to CI and tissue perfusion indices (SvO_2_, lactate) underscores the disadvantage of this index to reflect the impact of RV function on global hemodynamics.

### 3.5. Correlations of RV and LV Function

In order to investigate the interaction of RV and LV function including the Impella, we performed a Spearman correlation analysis between LVSWI, as a left ventricular performance indicator, and PAPi, RVSWI, and RA/PCWP. It should be mentioned that because LVSWI is a calculated value ((LVSWI) = SVI × (MAP − PCWP) × 0.0136), we considered LVSWI as a performance indicator of the “left ventricular system” (Impella + LV function), not of the LV. The analysis revealed significant positive correlations of LVSWI with PAPi (*p* < 0.001, correlation coefficient 0.275) and RVSWI (*p* < 0.001, correlation coefficient 0.423) and a significant negative correlation between LVSWI and RA/PCWP (*p* = 0.003, correlation coefficient −0.190) (Table 5). These results highlight the interdependence of RV and LV function during LV Impella support, once again underlining the relevance of RV function and the need for adequate monitoring of both ventricles.

## 4. Discussion

The treatment of CS patients is challenging, and mortality has remained consistently high in recent decades [22,23,24]. LV systolic failure is the pathology that most often determines the clinical situation. Therefore, LV mechanical cardiac assist devices, such as the Impella microaxial pump, have been increasingly used to support the hemodynamics of CS patients through LV unloading, thereby promoting myocardial recovery and providing additional cardiac output for improved end-organ perfusion and function. However, an improvement of LV function, which may primarily depend on the recovery of the myocardium and secondarily depend on myocardial unloading, does not necessarily lead to an improvement of RV function. The acknowledgment of the interference and communication between both ventricles is of utter importance.

However, in everyday clinical practice, it is often underrecognized that RV systolic function is almost as relevant as LV function regarding the prognosis. In particular, RV function depends on RV pre- and afterload, which during LVAD therapy, in certain cases and situations (e.g., due to geometric changes of the ventricles or left ventricular compression in situations of RV failure), increases significantly, as previously described. A similar situation may also occur during temporary mechanical hemodynamic support with the Impella, if volume status is not sufficient for the selected Impella performance level, resulting in suction and hemolysis.

In order to ensure the best possible treatment of CS and the most optimal Impella support, appropriate monitoring of RV function appears to be essential [25,26,27]. Josiassen et al. recently showed that the LV Impella can benefit RV function in cases of myocardial infarction-related CS in pigs [13]. However, to our knowledge, there is little human data examining the actual effect of different performance levels of the LV Impella on RV function and the interference of both ventricles during support. Furthermore, the evaluation and definition of the most optimal predictor for monitoring RV function during LV Impella support in daily clinical practice should be determined.

Thus, we herein compared three established RV function indices, i.e., PAPi, RVSWI, and RA/PCWP, evaluating the effect of the Impella on RV function. Moreover, we demonstrated the biventricular interference on various pre-defined Impella performance levels, which may help to better understand hemodynamics and therapy requirements that need to be observed during LV Impella therapy.

All indices of RV function were significantly associated with CI, highlighting the interaction of RV and LV systolic function. Furthermore, all three indices were significantly associated with CVD, as should be expected, since venous congestion burdens the RV with additional preload, deteriorating its systolic function. Vice versa, a worsening RV systolic function can lead to an increase in CVD.

As far as the PCWP is concerned, the PAPI and RA/PCWP were closely linked to its changes, whereas the RVSWI was not. This highlights PAPi and RA/PCWP as more appropriate to reflect the influence of the RV function on post-capillary congestion, burdening the LV.

Furthermore, PAPi was strongly associated with SvO_2_. RVSWI also showed a significant association with SvO_2_, though less significant than PAPi. Regarding RA/PCWP, no significant interaction with SvO_2_ was revealed. On the other hand, RVSWI was significantly associated with lactate, whereas PAPi did not show any relevant correspondence to lactate. Interestingly RA/PCWP was also significantly associated with lactate. In total, we observed a stronger association of RVSWI and PAPi to alterations of tissue perfusion-related values like SvO_2_ and lactate.

These results indicate that PAPi allows a comprehensive assessment of RV function, as it is significantly associated with global hemodynamics (CI), parameters of venous congestion (CVD), and post-capillary congestion (PCWP), as well as with tissue perfusion (SvO_2_). Moreover, according to the current literature, PAPi is accompanied by a higher predictive ability for major adverse cardiac events than other right ventricular function indices [18].

Regarding the influence of the LV Impella on RV function, the data of this study did not reveal any additional burden for the RV, as one may assume due to additional RV afterload. Augmenting Impella output did not lead to a significant variation of PAPI, RVSWI, or RA/PCWP. The prerequisite is an adequately selected performance level of the Impella with sufficient ventricular volume filling, thus avoiding suction with consequent variation of right ventricular morphology or influence on ventricular performance, depending on the Frank–Starling mechanism.

Moreover, the positive association of PAPi with CO, PCWP, CVD, and SvO_2_ underscores the importance of RV function. Therefore, the RV function in CS patients with Impella support should be routinely monitored, and therapy should be appropriately adjusted in order to optimize RV function and thus the prognosis of the patients.

This study has limitations. One is the relatively small number of patients and the experience of a single center, which limits the interpretation of the data. Furthermore, the short-term design does not allow conclusions regarding clinical outcomes and survival. However, this is one of only a few human studies investigating the effect of the Impella on RV function. In order to further analyze the clinical relevance of these findings, future prospective randomized studies with larger cohorts are necessary.

## 5. Conclusions

Although the clinical focus in CS is on monitoring LV function, adequate monitoring of RV function is essential during therapy. RV dysfunction or failure leads to a decrease in left ventricular preload and function, thus further worsening the hemodynamic situation. RV failure is a feared complication during LVAD therapy. However, the impact of percutaneously implanted temporary MCS, like the left ventricular Impella, on RV function has not been described before. Additionally, there is currently no recommended parameter for monitoring the RV function during Impella support. According to our recent data, no relevant impairment of RV function could be demonstrated during left ventricular Impella support. Furthermore, among the commonly used parameters for monitoring RV function, PAPi appears to have the most comprehensive ability to reflect hemodynamic changes during Impella support in CS.

## Figures and Tables

**Table 1:** Demographics, baseline characteristics, and comorbidities. (BMI: body mass index, CHD: coronary heart disease, LVEF: left ventricular ejection fraction, TAPSE: tricuspid annular plane systolic excursion, TKS’ lateral: lateral tricuspid annular tissue doppler pulse wave velocity, IVC: inferior vena cava).

Demographics, Characteristics, and Comorbidities	
Age (years)	67 ± 13
Female (%)	26
Male (%)	74
1-vessel CHD (*n*)	19
2-vessel CHD (*n*)	13
3-vessel-CHD (*n*)	18
Chronic obstructive pulmonary disease (*n*)	9
Pulmonary artery hypertension (*n*)	0
BMI	26.4 ± 3.4
Impella flow (min/max L/min)	1.5/2.5 L/min ± 0.4 L/min
LVEF at Impella implantation (%)	37 ± 18
Mechanical ventilation	*n* = 35
Pulmonary end-expiratory pressure (mmHg)	8.6 ± 2.01
Peak inspiratory pressure (mmHg)	21.62 ± 4.74
TAPSE (cm) baseline	20.34 ± 4.19
TKS’ lateral baseline	11.58 ± 2.27
Diameter of IVC (cm) baseline	1.88 ± 0.54

**Table 2:** Hemodynamics and dosages of catecholamines during different Impella flow levels. DAP: diastolic arterial pressure, SAP: systolic arterial pressure, MAP: mean arterial pressure, HR: heart rate.

Impella Output (IO) (L/min)	Baseline (BL)	+0.5 L/min	+1.0 L/min
DAP (mmHg)	59.61 ± 11.1	59.88 ± 11.0	60.22 ± 11.9
SAP (mmHg)	110.9 ± 18.1	111.1 ± 18.8	111.53 ± 18.9
MAP (mmHg)	85.6 ± 12.1	86.1 ± 12.7	89.1 ± 12.8
HR (bpm)	100.18 ± 13.3	101.26 ± 15.05	101.96 ± 14.73
Norepinephrine (µg/kg/min)	0.12 ± 0.19	0.12 ± 0.19	0.12 ± 0.19
Dobutamine (µg/kg/min)	3.1 ± 2.67	3.1 ± 2.67	3.1 ± 2.67

**Table 3:** Change of right ventricular function indices and tissue perfusion parameters on various Impella output levels. IO: Impella output, PAPi: Pulmonary Artery Pulsatility index, RVSWI: right ventricular stroke work index, RA/PCWP: right atrial pressure/pulmonary capillary wedge pressure, SvO_2_: central venous oxygen saturation. * Variables displayed as mean ± SD. ^‡^ Variables displayed as median (interquartile range).

Impella Output (IO) [L/min]	Baseline (BL)	+0.5 L/min	+1.0 L/min	*p*-Value
_log_PAPi	1.248 ± 0.569	1.268 ± 0.632	1.353 ± 0.01	0.717
PAPi ^‡^	2.033 (2.01)	2.0 (2.63)	2.19 (2.84)	
PAPi *	3.286 ± 3.801	3.538 ± 4.2	4.94 ± 7.91	
_log_RVSWI	1.95 ± 0.69	1.86 ± 0.7	2.01 ± 0.74	0.599
RVSWI ^‡^	6.94 (5.71)	6.75 (6.03)	6.92 (6.81)	
RVSWI *	7.77 ± 5.86	6.92 ± 4.51	8.42 ± 6.72	
_log_RA/PCWP	0.613 ± 0.343	0.609 ± 0.377	0.55 ± 0.29	0.601
RA/PCWP ^‡^	0.785 (0.5)	0.767 (0.47)	0.75 (0.5)	
RA/PCWP *	0.976 ± 0.903	1.02 ± 1.262	0.815 ± 0.588	
SvO_2_ (%) ^‡^	63.9 (17.0)	65.9(14.3)	66.9 (15.5)	0.498
SvO_2_ (%) *	62.15 ± 14.9	65.5 ± 10.82	67.19 ± 10.44	
Lactate (mmol/L) ^‡^	1.2 (1.0)	1.2 (0.85)	1.2 (0.75)	0.112
Lactate (mmol/L) *	1.71 ± 1.61	1.55 ± 1.46	1.54 ± 1.32	

**Table 4:** Linear regression analysis of parameters with predictor _log_-variables. CI: cardiac index, PCWP: pulmonary capillary wedge pressure, CVD: central venous pressure, PAPi: Pulmonary Artery Pulsatility index, RVSWI: right ventricular stroke work index, RA/PCWP: right atrial pressure/pulmonary capillary wedge pressure, SvO_2_: central venous oxygen saturation.

Predictor Variable: _log_PAPi			
	*p* value	regression–coefficient B	95% CI
CI	<0.001	0.275	0.181–0.663
PCWP	0.005	−0.231	−6.721–−1.26
CVD	<0.001	−0.687	−7.89–5.575
SvO_2_	0.008	0.218	1.096–7.196
lactate	0.686	0.033	−0.276–0.419
**Predictor Variable: _log_RVSWI**			
	*p* value	regression–coefficient B	95% CI
CI	<0.001	0.426	0.409–0.841
PCWP	0.406	0.068	−1.54–3.8
CVD	0.03	−0.181	−3.19–−0.201
SvO_2_	<0.001	0.340	3.21–8.55
lactate	<0.001	−0.333	−0.992–−0.366
**Predictor Variable: _log_RA/PCWP**			
	*p* value	regression–coefficient B	95% CI
CI	0.096	−0.06	−0.234–−0.104
PCWP	<0.001	−0.301	−5.624–−1.753
CVD	<0.001	0.234	1.39–7.337
SvO_2_	0.96	0.043	−4.317–7.551
lactate	0.042	−0.221	−1.618–−0.293

**Table 5:** Spearman correlation between LVSWI and PAPi, RVSWI, and RA/PCWP. LVSWI: left ventricular stroke work index, PAPi: Pulmonary Artery Pulsatility index, RVSWI: right ventricular stroke work index, RA/PCWP: right atrial pressure/pulmonary capillary wedge pressure.

LVSWI		
	correlation–coefficient (r_s_)	*p* value
PAPi	0.275	*p* < 0.001
RVSWI	0.423	*p* < 0.001
RA/PCWP	−0.190	*p* = 0.003

## Data Availability

Data are available upon individual request.
